# Regiospecific Positioning of Palmitic Acid in Triacylglycerol Structure of Enzymatically Modified Lipids Affects Physicochemical and In Vitro Digestion Properties

**DOI:** 10.3390/molecules26134015

**Published:** 2021-06-30

**Authors:** Hyeon-Jun Chang, Jeung-Hee Lee

**Affiliations:** Department of Food and Nutrition, Daegu University, Gyeonsan 38453, Korea; chj931116@naver.com

**Keywords:** in vitro digestion, palmitic acid, triacylglycerol, physicochemical property

## Abstract

Tripalmitin-(PPP, 81.2%), 1,3-dipalmitoyl-2-oleoylglycerol-(POP, 64.4%), 1,2-dipalmitoyl-3-oleoylglycerol-(PPO, 86.5%), and 1,3-dioleoyl-2-palmitoylglycerol-(OPO, 50.2%)-rich lipids with different regiospecific positions of palmitic acid (P) were synthesized via acetone fractionation and lipase-catalyzed acidolysis, and their physicochemical and hydrolytic characteristics were compared. Triacylglycerols (TAGs) with higher content of P, wherein P was at the *sn*-1 (or 3) position, had higher melting points, crystallization temperatures, and packing densities of fat crystals compared to those with a lower content of P, and with P at the *sn*-2 position. The in vitro digestion degree calculated as released fatty acid (FA) (%) at 30, 60, and 120 min was in the following order: OPO-rich > PPO-rich > POP-rich lipids. At 120 min, in vitro digestion of the OPO-rich lipid released 92.6% of fatty acids, resulting in the highest digestibility, while 89.7% and 87.2% of fatty acids were released from the OPO-rich and PPO-rich lipids, respectively. Over the digestion period, the TAG and monoacylglycerol (MAG) contents decreased, while the diacylglycerol (DAG) content initially increased and then decreased, and the 1,2-DAG content exceeded the 1,3-DAG content. Therefore, the content and stereospecific position of P attached to a specific TAG affected the physicochemical and in vitro digestion characteristics of the lipids.

## 1. Introduction

Triacylglycerol (TAG) is a lipid class consisting of one glycerol molecule linked to three fatty acids (FAs) via ester bonds. The type and regiospecific position of FAs incorporated into glycerol molecules affect the physicochemical and digestion characteristics of TAGs, such as the TAG profile, solid fat content, polymorphism, crystal morphology, melting point, and melting and crystallization temperatures [1]. Palmitic acid (P) is a major FA in palm oil, palm stearin, cocoa butter, and lard, and 1,2-dipalmitoyl-3-oleoylglycerol (PPO), a major P-containing TAG, was found in these lipids as 31.61%, 15.23%, 18.08%, and 10.63%, respectively [2]. Notably, 1-oleoyl-2-palmitoyl-3-linoleoylglycerol (OPL 28.08%) and 1,3-dioleoyl-2-palmitoylglycerol (OPO 19.5%) are the major TAGs found in breast milk [3]. Differential scanning calorimetry (DSC) has shown that the melting peaks of 1,3-dipalmitoyl-2-oleoylglycerol (POP) and OPO are 36.5 and 21.9 °C, respectively [4,5]. Crystal morphology of OPO is characterized based on the evenness and density distribution of crystals in a small Maltese cross [6]. Notably, 1,2-dipalmitoyl-3-stearoylglycerol (PPS) and 1,3-dipalmitoyl-2-stearoylglycerol (PSP) are found in the Maltese cross form at −25 °C [7]. TAGs produced using higher P and lower stearic acid (S) contents generally contain more β’ form than β-form crystals [8].

TAGs have different digestion, absorption, and bioavailability rates depending on the type of FAs (unsaturation and carbon length) and the positions (*sn*-1,3 or *sn*-2) of the incorporation of FAs [9]. After ingestion, lipids are emulsified by bile acids and the FAs in the *sn*-1,3 position are hydrolyzed by pancreatic lipases, followed by absorption of 2-monoacylglycerols (MAGs) and free fatty acids (FFAs) in enterocytes [9,10]. Long-chain saturated FAs (14~20 carbons in chain length), such as P and S, in the *sn*-1,3 position of TAGs can be excreted as a form of insoluble FA soap after being hydrolyzed and bound with calcium or magnesium ions [9,10]. Among FAs, short-chain FAs (4~6 carbons in chain length) are most rapidly broken down by pancreatic lipases, followed by long-chain/unsaturated FAs and saturated FAs [9]. Based on the apparent digestibility of individual FAs following the administration of soybean oil to mice, the digestibility of P (88.43%) was higher than that of S (85.87%) in saturated FAs (*p* < 0.05), while the digestibility of linolenic acid (96.2%) was the highest in unsaturated FAs, followed by those of linoleic acid (93.63%) and oleic acid (91.53%) (*p* < 0.05) [11]. The in vivo absorption rates of the OPO and OOP diets in rats have been reported to be 80% and 67%, respectively, and as an individual FA, the absorption rates of P in the administrated OPO and OOP are 80% and 63%, respectively [12]. However, the results available on the importance of FA position on TAG digestion are still limited.

Tailored TAGs with P at specific locations (*sn*-1,3 or *sn*-2) were synthesized by solvent fractionation or enzyme-catalyzed interesterification. For example, PPP-rich and POP-rich lipids were synthesized from palm stearin by acetone-fractionation [13,14], OPO (39.2%) was synthesized with PPP-rich lipid, oleic acid, and linoleic acids by enzyme-catalyzed interesterification [15]; cocoa butter replacer (PPP, POO/OPO, POP/PPO) was produced with coconut oil and palm stearin by enzyme-catalyzed reaction [16]; and OPO was synthesized with tripalmitin (PPP) and oleic acid by lipase-catalyzed acidolysis [17]. Most studies have focused on analyzing the physicochemical properties of TAGs that differ in the regiospecific positions of certain FAs [13,14,15,16,17]. Previous studies on lipid digestibility have measured and compared the relative digestion rates of edible oils (soybean oil, algae oil, DAG-rich algae oil) [18] and symmetrical or non-symmetrical TAGs based on the levels of hydrolyzed FFAs using the in vitro digestion method [19] or compared in vivo digestion rates of specific edible oils using animal models [11]. However, studies comparing the digestion characteristics of lipids containing TAGs with different types of FAs (such as P, O, or S) and the incorporated positions of the FAs are still limited.

To study lipid digestion, two in vitro digestion models are conveniently used. A pH stat-based model provides time-dependent kinetics of lipid lipolysis during a short period of digestion, and is useful as a rapid screening tool for assessing lipid digestibility; however, this model is a single-step model that simplifies the human digestive process by matching the chemical composition and pH of digestive condition in the small intestine only. On the other hand, the in vitro multi-step digestion model is designed to simulate the complex digestive process of the entire human gastrointestinal tract of the mouth, stomach, small intestine, and colon with a specifically composed digestive fluid (i.e., pH, mineral composition, enzymes) [20]. Since the multiple-step digestion model proceeds the entire digestibility step by step, it can provide the status of lipid hydrolysis at specific points of lipid digestion, and studies can investigate the behavior of lipid digestion by measuring the composition of fatty acids and acylglycerols and their contents.

Up to date, most in vitro multi-step digestion models for lipids have been performed with pancreatic lipase in the small intestine, excluding the gastric lipase in the gastric phase. Sams et al. [21] noted that out of 340 articles published from 1967 to 2015, 94.9% of the total in vitro models did not include gastric lipase since the lipase activity is markedly lower in the gastric phase than in the duodenal tract, and the lipolysis of gastric lipase is partial because of the lower secretion and variation in pH [22]. However, lipid digestion starts in the stomach with gastric lipase on TAGs, and gastric lipolysis contributes to the overall lipid digestion by triggering the subsequent action of pancreatic lipase on lipids. Therefore, recently, the amended and improved in vitro gastrointestinal digestion method (INFOGEST 2.0) included the use of gastric lipase in the gastric phase [23]. There are several commercial gastric lipases, such as rabbit gastric lipase and human gastric lipase encoded by the LIPF gene, but the high cost of these lipases can be a limiting factor for performing the in vitro gastrointestinal digestion model.

In this study, four lipids were synthesized to assess the physicochemical and digestion characteristics of TAGs varying in the regiospecific position of palmitic acid. PPP-rich and POP-rich lipids were obtained via acetone fractionation of palm stearin, while PPO-rich and OPO-rich lipids were synthesized via lipase-catalyzed acidolysis. The physicochemical properties of the PPP-rich, POP-rich, PPO-rich, and OPO-rich lipids were compared by analyzing the compositions of acylglycerol, TAGs, and FAs; melting and crystallization properties; and crystal morphologies. Additionally, the digestion characteristics of the POP-rich, PPO-rich, and OPO-rich lipids were assessed by measuring their FA hydrolysis rates using an in vitro multi-step digestion model and by analyzing the changes in the acylglycerol and TAG compositions of the hydrolyzed lipids over digestion time.

## 2. Results and Discussion

### 2.1. Acylglycerol Composition

**Figure 1:**
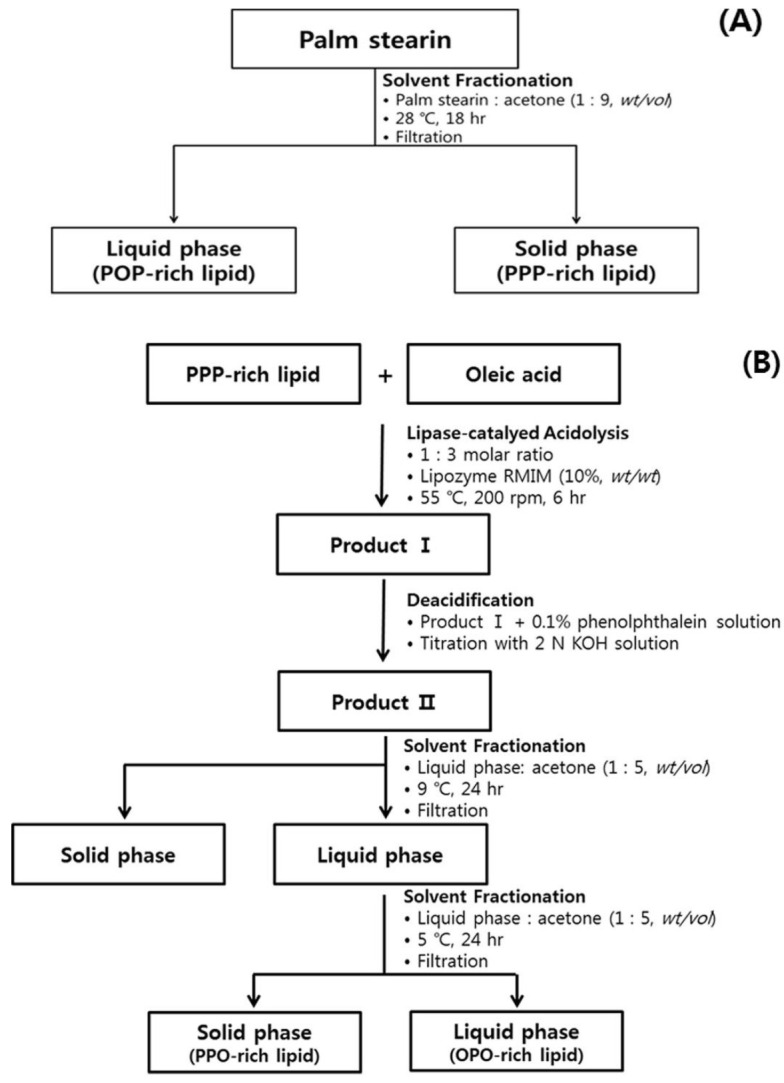
Production scheme of PPP-rich and POP-rich lipids (**A**), PPO-rich, and OPO-rich lipids (**B**).

The acylglycerol compositions of the four lipids with different regiospecific positions of palmitic acid analyzed by ^1^H-NMR, and the composition are presented with mmol% using a tetramethylsilane (TMS) as a reference in Table 1. Acetone fractionation of palm stearin consisting of TAG (96.9 mmol%) and DAG (3.1 mmol%) at 28 °C produced PPP-rich lipid, which was in the solid phase and consisted entirely of TAG (100 mmol%), and POP-rich lipid, which was in the liquid phase and consisted of 93.4 mmol% TAG, 6.3 mmol% DAG, and 0.3 mmol% MAG. The presence of DAG and MAG in the POP-rich lipid may be attributed to their relatively low melting points, which contributed to the transition into the liquid phase during the fractionation process (Figure 1). The PPO-rich and OPO-rich lipids consisted of 99.1~99.3 mmol% TAG, 0.7 mmol% DAG, and 0.1~0.2 mmol% MAG.

### 2.2. Fatty Acids Composition

The major FAs in palm stearin were P (C16:0, 63.4%), O (C18:1, 24.9%), L (C18.2, 5.1%), and S (C18:0, 4.8%), and the main FAs at the *sn*-2 position were P (45.6%), O (42.5%), and L (9.1%) (Table 2 and Table 3). The major FAs in the four lipids obtained were P (30.2~79.4%), S (2.5~5.0%), O (11.6~60.9%), and L (2.12~8.19%) (Table 2). The P contents in the lipids were in the following order: PPP-rich (79.4%) > PPO-rich (46.9%) > POP-rich (46.5%) > OPO-rich lipids (30.2%) (*p* < 0.05). The O contents in the lipids were in the following order: OPO-rich (60.9%) > PPO-rich (45.3%) > POP-rich (38.9%) > PPP-rich lipids (11.6%) (*p* < 0.05). The S and L contents were significantly higher in the PPP-rich and POP-rich lipids, respectively (*p* < 0.05).

The P contents at the *sn*-2 position in the lipids were as follows: PPO-rich (81.2%) > PPP-rich (73.4%) > OPO-rich (60.3%) > POP-rich lipids (14.3%) (*p* < 0.05). The O contents at the *sn*-2 position in the lipids were as follows: POP-rich (67.86%) > OPO-rich (31.80%) > PPP-rich (19.91%) > PPO-rich lipids (14.3%) (*p* < 0.05) (Table 3). The P contents at the *sn*-1,3 position were as follows: PPP-rich (82.3%) > POP-rich (62.5%) > PPO-rich (29.7%) > OPO-rich lipids (15.2%) (*p* < 0.05). The O contents at the *sn*-1,3 position were significantly higher in the following order: OPO-rich (75.4%) > PPO-rich (61.4%) > POP-rich (24.3%) > PPP-rich lipids (7.5%) (*p* < 0.05). The four lipids synthesized in the present study contained 85.2–92.2% of P and O, and the incorporated positions were distinctly different on the glycerol backbones of TAGs.

### 2.3. Triacylglycerol Composition

**Figure 2:**
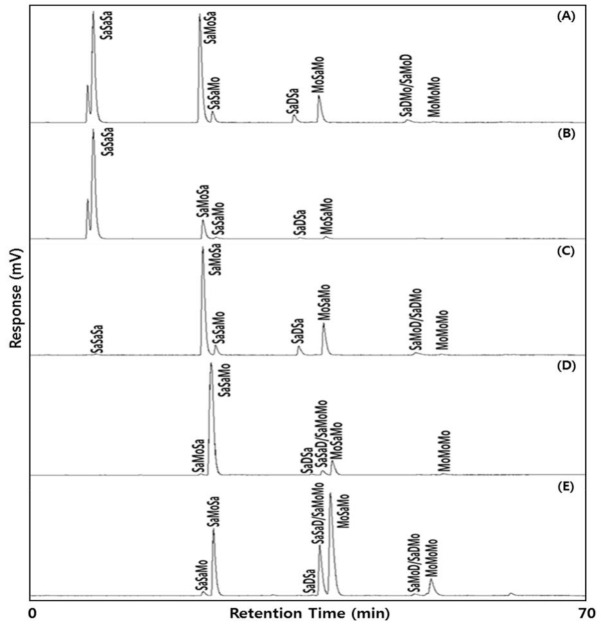
Ag-HPLC chromatograms of the lipids, (**A**) Palm stearin; (**B**) PPP-rich lipid; (**C**) POP-rich lipid; (**D**) PPO-rich lipid; (**E**) OPO-rich lipid. The abbreviations are presented in the results and discussion.

TAGs in the lipids were separated based on the number of double bonds and their geometrical configurations using silver ion-HPLC, and designated as saturated FA (Sa), monounsaturated FA(Mo), and diunsaturated FA(D) in the HPLC chromatogram (Figure 2). 

According to the results of the FA analysis (Table 2 and Table 3), Sa, Mo, and D were composed mainly of P, O, and L. Notably, Sa contained a small amount of S. Thus, the major TAGs were SaSaSa (PPP, PSP, SPS, PPS, SSP, SSS; main TAG, PPP), SaMoSa (POP, POS, SOS; main TAG, POP), SaSaMo (PPO, SSO, PSO, SPO; main TAG, PPO), MoSaMo (OPO, OSO; main TAG, OPO), and SaSaD/SaDSa (PPL, SSL, PSL, SPL/POO, SOO; main TAG, POO). The minor TAGs were SaDSa (PLP, PLS, SLS; main TAG, PLP), SaDMo/SaMoD (PLO, SLO/POL, SOL; main TAG, PLO/POL), and MoMoMo (OOO) (Table 4).

The major TAGs of palm stearin were PPP (38.0%), POP (42.4%), PPO (3.8%), PLP (3.2%), and OPO (10.8%). The major TAGs of the PPP-rich lipids were PPP (81.2%) and POP (15.1%), and those of the POP-rich lipids were POP (64.4%) and OPO (20.3%). The major TAGs of PPO-rich lipids were PPO (86.5%) and OPO (8.7%). The major TAGs of the OPO-rich lipids were OPO (50.2%), PPO (22.020%), and PPL/POO (18.6%), and a small amount of OOO (6.8%) also constituted OPO-rich lipids. Therefore, the four lipids synthesized mainly contained PPP, POP, PPO, and OPO, the contents of which varied. Wei et al. [17] produced a lipid consisting of 40.23% OPO by Lipozyme RMIM-catalyzed acidolysis, and Lee et al. [13] synthesized a lipid with 32.68% OPO from palm stearin and oleic acid using Lipozyme TLIM. The OPO content (50.2%) of the OPO-rich lipid in the present study was 1.25–1.54 times higher than in the previous studies.

### 2.4. Melting Points

The slip melting point (SMP) and complete melting point (CMP) of palm stearin were 55.6 and 57.0 °C, respectively (Table 2). The SMPs of the four lipids were in the following order: PPP-rich (59.5 °C) > POP-rich (27.3 °C), PPO-rich (26.5 °C) > OPO-rich lipid (15.0 °C) (*p* < 0.05). The CMPs were in the following order: PPP-rich (61.5 °C) > POP-rich (29.5 °C) > PPO-rich (27.5 °C) > OPO-rich lipid (18.5 °C) (*p* < 0.05). The MPs of lipids were influenced by their FA contents. The higher the SFA content, the higher the MP, and the higher the content of short-chain FA and USFA, the lower the MP [24]. Among the four lipids, the MP of PPP-rich lipid with the highest SFA content (86.0%) and the lowest USFA content (14.0%) was the highest, while that of the OPO-rich lipid with the lowest SFA content (33.5%) and highest USFA content (61.9%) was the lowest. The CMPs of POP-rich lipids were higher than those of PPO-rich lipids (*p* < 0.05) because although the SFA contents in POP-rich (52.1%) and PPO-rich lipids (51.2%) were similar, their major SFAs were S (MP, 69.4 °C) and P (MP, 62.5~63.0 °C), respectively, and the S content was 1.21 times higher in PPO-rich lipids than in POP-rich lipids (*p* < 0.05) [25,26,27]. The MPs of lipids were also affected by the locations of the incorporated FAs (*sn*-1,3 or *sn*-2) in the TAG structure. Motoyama [28] reported that the MPs of TAGs containing P, S, and O are higher for POP (37 °C) and SOS (43 °C) than for PPO (35 °C) and SSO (41 °C), respectively, and that the MP is increased when SFA is incorporated in the *sn*-1,3 position rather than the *sn*-2 position. Consistent with these reports, the MP of the POP-rich lipid was higher than that of the PPO-rich lipid due to the two-fold higher SFA content at the *sn*-1,3 position and a higher S content in the POP-rich lipid than in the PPO-rich lipid.

### 2.5. Crystallization and Melting Properties

**Figure 3:**
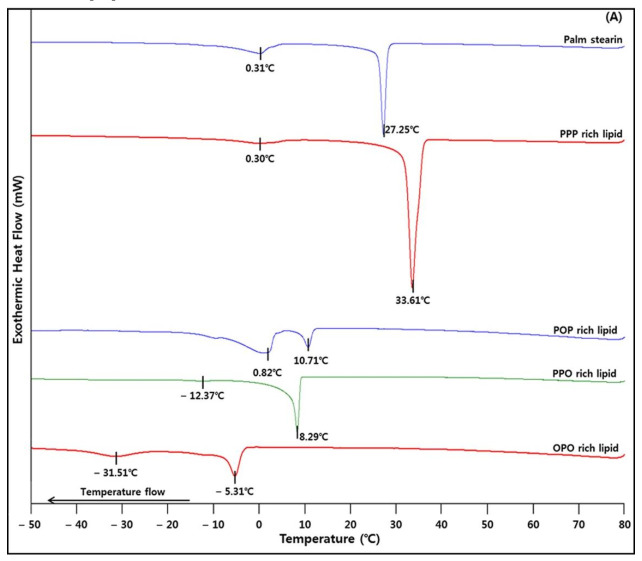

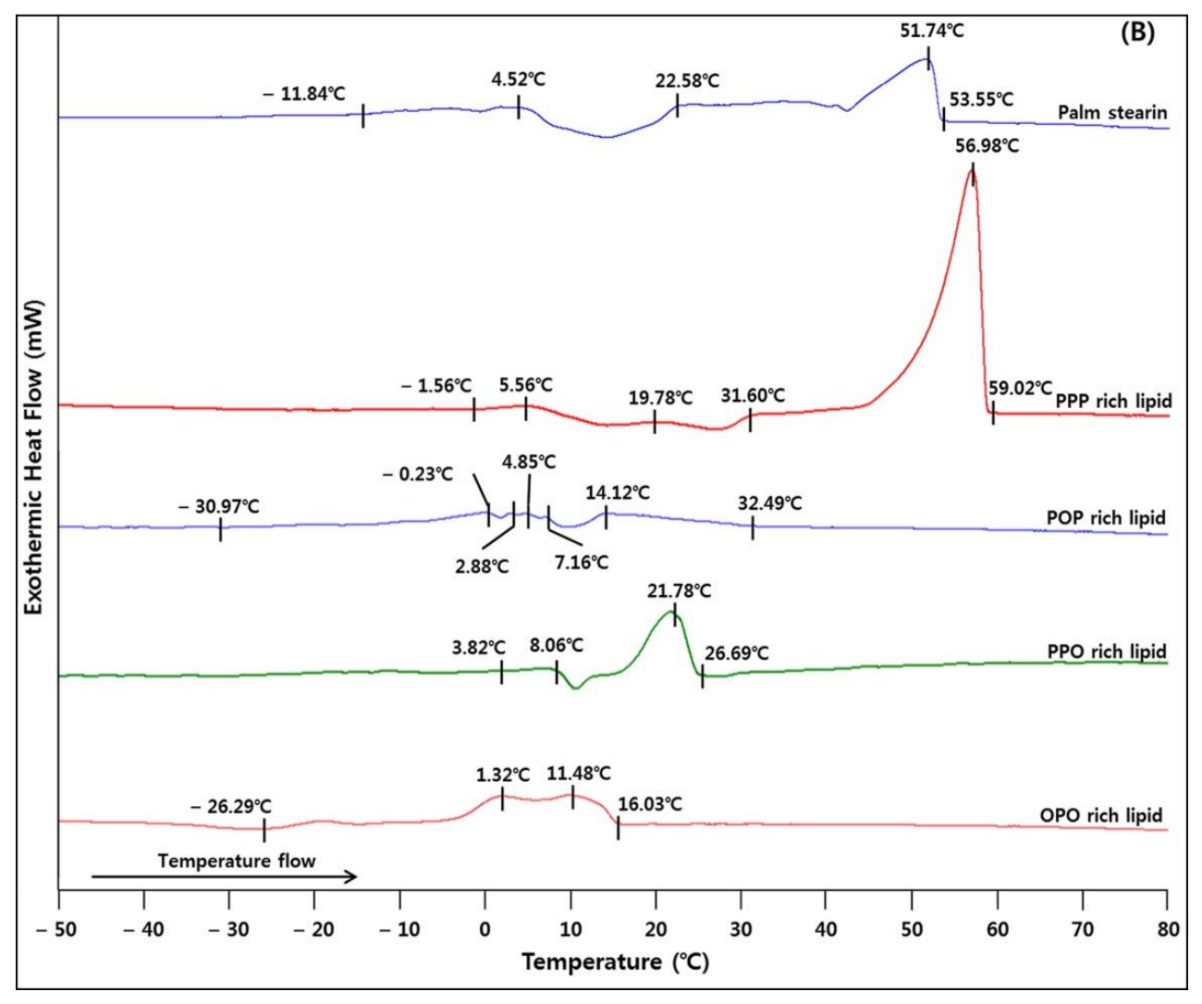
Differential scanning calorimetry crystallization (**A**) and melting (**B**) curves of the lipids.

The melting and crystallization characteristics analyzed by DSC are shown in Figure 3. In the crystallization curve (Figure 3A), palm stearin showed a large sharp crystallization peak at 27.25 °C and a small peak at 0.31 °C. The crystallization onset temperatures were in the following order: PPP-rich > POP-rich > PPO-rich > OPO-rich lipids. The PPP-, PPO-, and OPO-rich lipids showed very distinct large crystallization peaks at 33.61, 8.29, and −5.31 °C, respectively. Thus, it appears that crystallization mostly occurs at these temperatures. The POP-rich lipid showed two crystallization peaks (10.71 and 0.82 °C) from −19.08 °C to 12.62 °C, indicating that crystallization occurred twice in this temperature range. The four lipids had different crystallization onset temperatures and crystallization peaks due to their different TAG compositions and contents. Gwie et al. [29] reported a large crystallization peak at 38.8 °C for PPP (purity 85%), which is higher than the temperature at which a crystallization peak was observed (33.61 °C) for the PPP-rich lipid (SaSaSa, 81.2%) in the present study. Even though the SaSaSa concentration is lower than the PPP concentration (85%) [29], since SaSaSa in PPP-rich lipid was composed of a mixture of P (major) and S (minor), the crystallization peak of SaSaSa can be lower than that of PPP [29].

In the melting curve (Figure 3B), palm stearin showed a large melting peak at 51.74 °C, and completely melted at 53.55 °C. PPP-rich and PPO-rich lipids had large melting peaks at 56.98 and 21.78 °C, respectively, and melted completely at 59.02 and 26.29 °C, respectively. Although POP-rich and OPO-rich lipids did not show large melting peaks, their melting ranges were −30.92~32.49 and −26.29~16.03 °C, and these lipids melted completely at 32.49 and 16.03 °C, respectively. In the melting curves of palm stearin, PPP-rich, PPO-rich, and OPO-rich lipids, the DSC melting end points were in the range of SMP–CMP (Table 2), showing the consistency of the analyzed melting property between DSC and the capillary tube method (for measuring SMP and CMP).

### 2.6. Crystal Morphology

**Figure 4:**
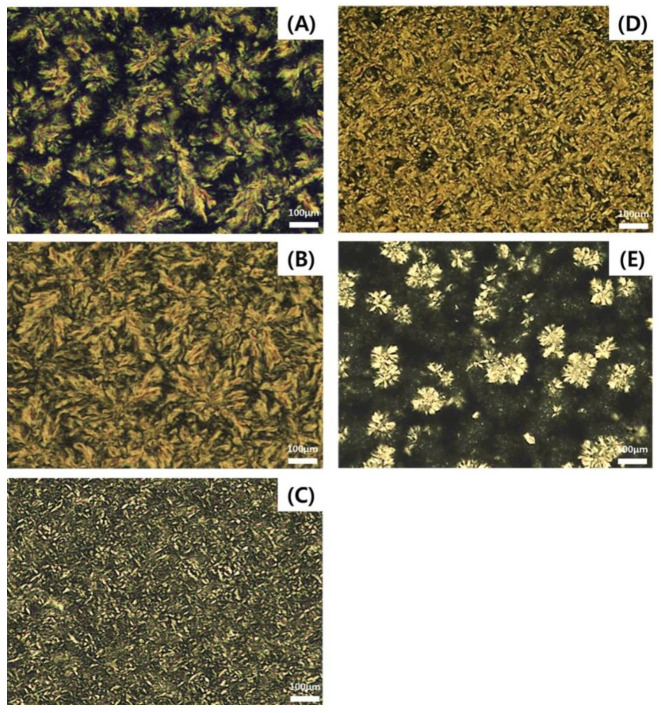
Polarized light micrographs of (**A**) palm stearin, (**B**) PPP-rich lipid, (**C**) POP-rich lipid, (**D**) PPO-rich lipid, and (**E**) OPO-rich lipid (stored at 4 °C for 16 h). The scale bar represents 100 μm.

Figure 4 shows the distinctly different crystal morphologies of palm stearin and PPP-rich, POP-rich, PPO-rich, and OPO-rich lipids stored at 4 °C for 16 h. Rod-like spherulitic crystals were observed for palm stearin, showing the similar crystal morphology in palm stearin of the study of Lee et al. [8]. The PPP-rich lipid with a high crystallization temperature had the largest crystals and a high packing density among all the lipids. The crystals in the POP-rich lipid were in the form of a small needle, the smallest in size, and highly packed. The PPO-rich lipid showed rod-like crystals that were larger and more tightly packed than the crystals in the POP-rich lipid. The OPO-rich lipid had small and large crystal aggregates in the shape of a spherical Maltese cross with low packing density. The OPO-rich lipid had a distinct crystallization peak at −5.31 °C, while it had a broad melting range of −5.44 to 16.03 °C with multiple melting peaks due to having a mixture of TAGs (Figure 3B), and melted completely at 18.5 °C (Table 2). It seemed that the various TAGs present in OPO-rich lipid were simultaneously crystallized and melted at the storage temperature of 4 °C for 16 h. During this process, crystal nucleation, growth, and agglomeration proceeded, leading to the formation of diverse crystal aggregates of clusters, in which the solid fat (white) was held in the liquid oil (black) (Figure 4E).

### 2.7. Free Fatty Acids (FFAs) Released by the Hydrolyzed Lipids during In Vitro Multi-Step Digestion

**Figure 5:**
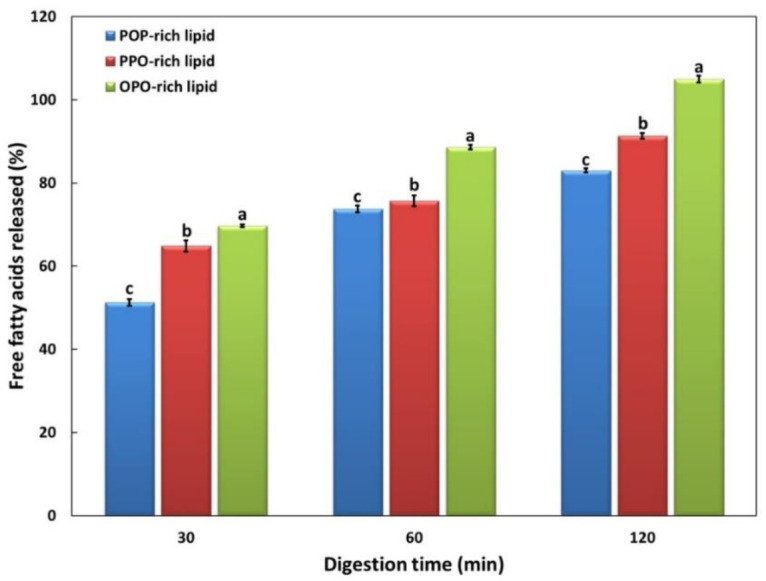
Free fatty acid released (%) by POP-rich lipid, PPO-rich lipid, and OPO-rich lipid during in vitro multi-step digestion model at 30, 60, and 120 min. ^a–^^c^ Means with different letters above the bars at the same digestion time are significantly different at *p* < 0.05 by Duncan’s multiple range test. Mean ± SD (*n* = 3).

The in vitro digestion degrees of POP-rich, PPO-rich, and OPO-rich lipids were measured based on the levels of FFAs at 30, 60, and 120 min of digestion using the in vitro multi-step model (Figure 5). The digestion degrees of all lipids increased with digestion time, and were in the following order: OPO-rich > PPO-rich > POP-rich lipids with a significance at each digestion time (*p* < 0.05). The FFAs released (%) of the OPO-rich lipid were 1.07 and 1.36 times higher at 30 min, 1.17 and 1.20 times higher at 60 min, and 1.15 and 1.27 times higher at 120 min than those of the PPO- and POP-rich lipids, respectively.

The pancreatic lipase used in the in vitro digestion model was *sn*-1,3 regiospecific lipase, which breaks down TAG into two FFA molecules and one 2-MAG molecule. *Sn*-1,3 regiospecific lipase has been reported to hydrolyze all FAs at the *sn*-1,3 position during digestion, but only 22% of FAs at the *sn*-2 position [10]. Lipids are hydrolyzed to different extents depending on their type (unsaturation and chain length) and location (*sn*-1,3, *sn*-2) of incorporating FAs. The hydrolysis rate has been reported to be higher for short-chain fatty acids (SCFAs) and USFAs than for long-chain fatty acids (LCFAs) and SFAs, respectively [9]. The hydrolysis rates of major FAs at the *sn*-1,3 positions were predicted to be high in the following order: O > P > S. In the present study, the OPO-rich lipids had high O contents and thus, higher levels of released FAs. Their hydrolysis rates were high in the following order: OPO-rich > PPO-rich > POP-rich lipids.

There is increasing evidence that the melting point affects the rate of lipid digestion. Steele and Moore [30] fed sheep a diet containing myristic acid (purity 95%), palmitic acid (96%), and stearic acid (94%) as lipid sources and measured their apparent digestibility based on the levels of secreted fecal lipids to compare the relationship between the melting point of each FA and its digestibility coefficient. They found that the higher the melting point of the FA, the lower the digestibility coefficient. Long-chain saturated FAs have low absorption coefficients as their melting points are higher than the body temperature and can show reduced digestibility as they form insoluble salts with calcium [10]. The CMPs of the POP-rich, PPO-rich, and OPO-rich lipids were 29.5, 27.5, and 18.5 °C, respectively, and the in vitro digestion degrees of these lipids increased as their melting points decreased.

### 2.8. Acylglycerol Composition of the Hydrolyzed Lipids during the In Vitro Multi-Step Digestion Model

**Figure 6:**
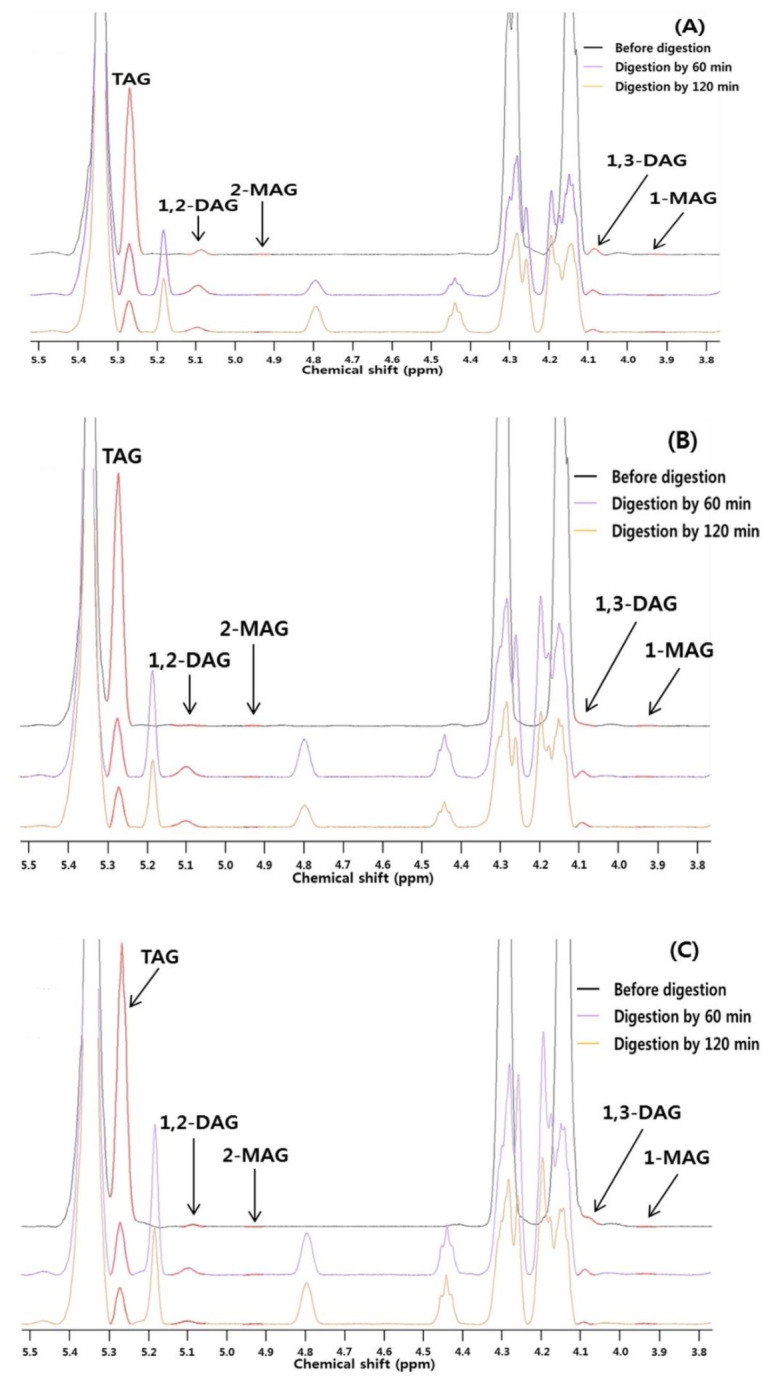
^1^H-NMR spectrum of POP-rich lipid (**A**), PPO-rich lipid (**B**), and OPO-rich lipids (**C**) during the in vitro multi-step digestion model. Expanded regions between 3.8 and 5.5 ppm. Before digestion (black line), in vitro digestion by 60 min (purple line), and in vitro digestion by 120 min (orange line).

The acylglycerol compositions of the hydrolyzed POP-rich, PPO-rich, and OPO-rich lipids at 60 and 120 min of in vitro digestion were analyzed, quantified, and presented in μmol with TMS as a reference (Figure 6 and Table 5). Figure 6 shows the enlarged ^1^H-NMR spectra of POP-rich lipid, PPO-rich lipid, and OPO rich lipid at regions of between 3.8 to 5.5 ppm (signal of TAG: 5.27, 1,3-DAG: 4.09, 1,2-DAG: 5.08, 1-MAG: 3.94, 2-MAG: 4.93 ppm) [31,32,33,34]. The TAG content steadily and rapidly decreased, while the MAG and DAG contents increased at 60 min but decreased thereafter.

The PPO-rich lipid consisted of TAG (848.12 μmol), DAG (6.12 μmol), and MAG (1.58 μmol). The TAG content significantly and rapidly decreased during in vitro digestion, and 15.33% and 11.91% of the TAGs remained at 60 and 120 min compared with before digestion, respectively (*p* < 0.05). The DAG and MAG contents increased after 60 min of in vitro digestion and decreased at 120 min. The DAG and MAG contents significantly increased by 7.4- and 1.8-fold at 120 min (*p* < 0.05). The 1,3-DAG content was 1.6 times higher than the 1,2-DAG content before digestion; however, the 1,2-DAG content significantly increased to 3.2- and 2.7-fold that of the 1,3-DAG content at 60 and 120 min (*p* < 0.05). The 1-MAG content was 3.8-fold higher than the 2-MAG content before digestion and 1.7 and 1.4 times higher than the 2-MAG content at 60 and 120 min, respectively.

The TAG content rapidly decreased in POP-rich lipids, resulting in 19.53% and 12.10% of the TAGs remaining at 60 and 120 min of in vitro digestion, respectively. The DAG content increased at 60 min and then decreased at 120 min (*p* < 0.05), whereas the MAG content consistently increased (*p* > 0.05). The 1,3-DAG content was 1.1-fold higher than the 1,2-DAG content before digestion, but the 1,2-DAG content increased to 3.4- and 2.7-fold higher than the 1,3-DAG content at 60 and 120 min, respectively. The TAG content of the OPO-rich lipid significantly decreased during digestion, resulting in 13.86% and 10.21% of the TAGs remaining at 60 and 120 min, respectively (*p* < 0.05). The DAG and MAG contents increased at 60 min and decreased at 120 min (*p* < 0.05). The 1,2-DAG content was 2.3- (at 60 min) to 3.7-fold (at 120 min) higher than the 1,3-DAG content. The 1-MAG content was 1.7-fold higher than the 2-MAG content at 60 min and similar at 120 min.

TAGs are hydrolyzed by pancreatic lipase (*sn*-1,3 position-specific enzyme) into 1,2-DAG and FFA, and 1,2-DAG is then hydrolyzed into 2-MAG and FFA; these two processes occur simultaneously. During lipid digestion over time, TAGs, which had the highest levels among the acylglycerols, were mainly degraded at 60 min, resulting in a transient increase in the levels of DAGs and MAGs. At 120 min, the produced DAGs and MAGs and the remaining TAGs were further hydrolyzed, leading to a consistent reduction in their levels and an increase in the FFA content. Since the hydrolysis rates of 1,3-DAGs and 1-MAGs were higher than those of 1,2-DAGs and 2-MAGs, respectively, due to the *sn*-1,3 specificity of pancreatic lipase, the levels of 1,3-DAGs and 1-MAGs decreased more significantly than those of 1,2-DAGs and 2-MAGs. The change of acylglycerol composition of in vitro digested sunflower and soybean oils showed a similar result of this study. Nieva-Echevarría et al. [34] reported that the TAG content of sunflower oil decreased after digestion compared to before digestion, and that the 1,2-DAG content increased and then decreased during digestion. Martin-Rubio et al. [35] reported that, after in vitro digestion of soybean oil, the TAG content decreased, while the 1,2-DAG and 1,3-DAG contents increased, and that the 1,2-DAG content was higher than the 1,3-DAG content, showing a similar trend to the results of this study.

The concentrations of the remaining TAGs at 60 and 120 min of digestion were in the following order: POP-rich > PPO-rich > and OPO-rich lipids. Therefore, it was predicted that the digestion degree would be high in the order of OPO-rich > PPO-rich > and POP-rich lipids, similar to the released FFA (%) (Figure 5).

### 2.9. Triacylglycerol Compositions of the Hydrolyzed Lipids during In Vitro Multi-Step Digestion

**Figure 7:**
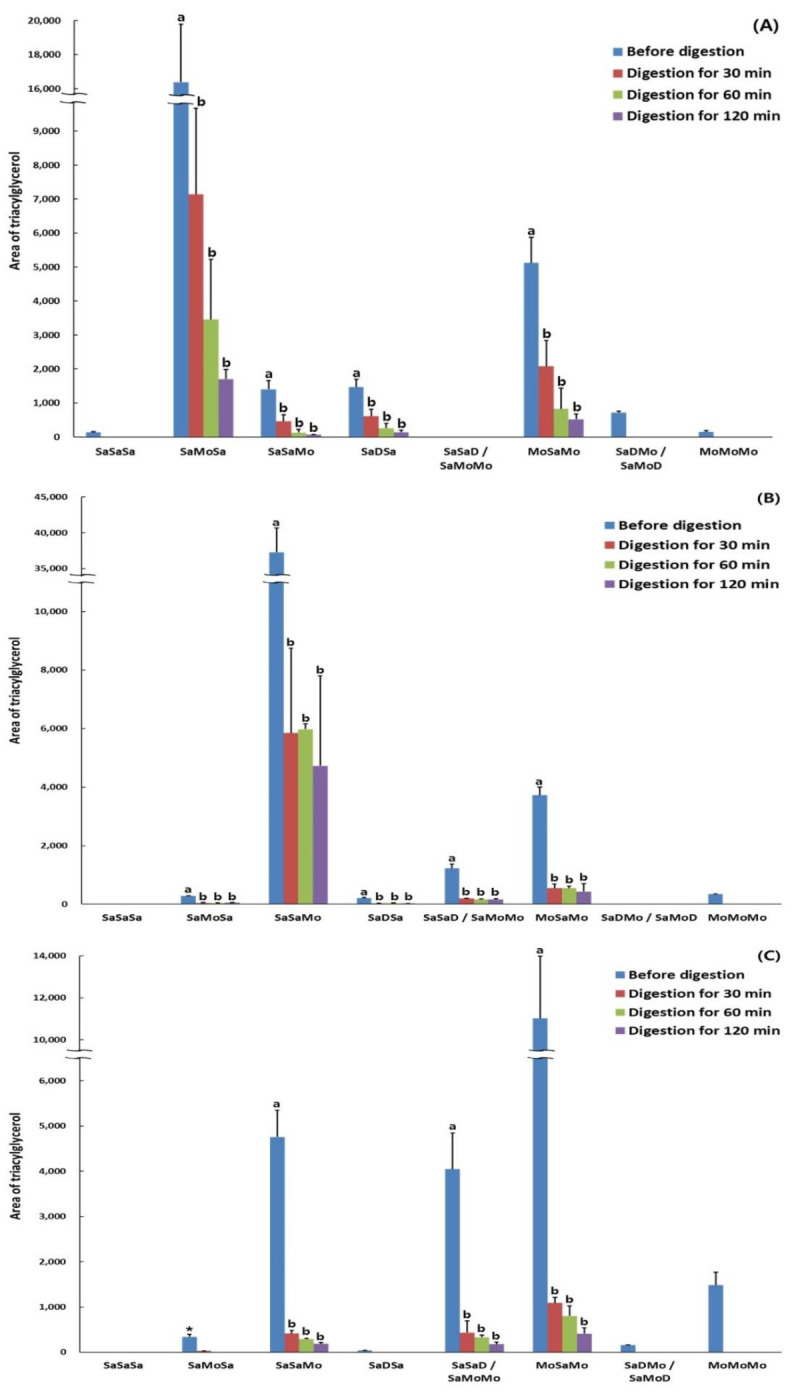
The major triacylglycerol compositions of POP-rich (**A**), PPO-rich (**B**), and OPO-rich lipids (**C**) determined during in vitro multi-step digestion analyzed with Ag-HPLC. ^a,b^ Means with different letters above the bars at the same digested lipids are significantly different by Duncan’s multiple range test (*p* < 0.05). * Differences between before digestion and 30 min-digested lipids are significantly different by Student’s *t*-test (*p* < 0.05). Mean ± SD (*n* = 3).

Each TAG of the hydrolyzed lipids rapidly decreased at 30, 60, and 120 min of in vitro digestion, whereas FFAs increased (Table 6 and Figure 7). OPO (MoSaMo), PPO (SaSaMo), and POP (SaMoSa), which are the major TAGs of OPO-rich, PPO-rich, and POP-rich lipids, respectively, decreased with digestion time, and the concentrations of the remaining TAGs were high in the following order: POP (43.53%) > PPO (15.79%) > OPO (9.87%) at 30 min, and POP (20.97%) > PPO (16.12%) > OPO (7.29%) at 60 min. This showed that the digestion degree of OPO was the highest, followed by those of PPO and POP. With a longer digestion time of 120 min, the OPO content remained at 3.73%, indicating that most of the OPO was hydrolyzed at the highest digestion degree (96.3%), whereas the remaining PPO and POP contents at 120 min were 12.76% and 10.34%, respectively, showing a greater increase in the digestion degree of POP than that of PPO between 60 and 120 min of digestion.

Since the FA hydrolysis rate by pancreatic lipase was high in the order of O > P > S, the rate of digestion for TAGs was predicted to be high in the following order: OPO > PPO > POP. However, POP showed a greater increase in the digestion degree compared to PPO at a digestion time of 120 min, indicating that the digestion degree consistently increased over time as hydrolysis occurred, even if the initial digestion degree was low (digestion rate, 60 min → 120 min; POP, 79.0% → 89.7%; PPO, 83.9% → 87.2%). Similar to the results of this study, a previous study investigating the in vitro hydrolysis rate of triolein (TO), tridocosahexaenoyl glycerol (TriDHA), and trieicosapentaenoyl glycerol (TriEPA) by pancreatic lipase reported that during the initial digestion within 10 min, more than 70% of TO was hydrolyzed rapidly, whereas TriDHA and TriEPA were hydrolyzed slowly by about 30% but increased markedly afterwards. In the end, most of the TriEPA and 80% of TriDHA and TO were hydrolyzed, and TriEPA showed a higher hydrolysis rate than TO [36].

## 3. Materials and Methods

### 3.1. Materials

Palm stearin was provided by Samyang Co., Ltd. (Seoul, Korea). Soybean oil was purchased from Ottogi Co., Ltd. (Pyeongteak-city, Korea). Lipase from porcine pancreas, oleic acid, bile salts, pancreatin from porcine pancreas, PPP standard, and Supelco 37 component FAME mix were purchased from Sigma-Aldrich Co., Ltd. (St. Louis, MO, USA). Lipozyme RMIM was purchased from Novozymes (Bagsvaerd, Denmark), and triundecanoin was purchased from NU-CHEK PREP, Inc. (Elysian, MN, USA).

### 3.2. Production of PPP-Rich and POP-Rich Lipids

PPP-rich and POP-rich lipids were prepared from palm stearin by acetone fractionation, as shown in Figure 1. Palm stearin was completely melted, mixed with acetone (1:9, *wt*/*vol*), and then placed at 28 °C for 18 h. The fractionated acetone mixture was separated into solid and liquid phases by filtration using filter paper (Whatman No. 4, GE Healthcare, Amersham, UK). The separated solid phase (PPP-rich lipid) and liquid phase (POP-rich lipid) were concentrated in a rotary vacuum evaporator (N-1110, Sunileyela, Seongnam-city, Korea) and then dried with N_2_.

### 3.3. Production of PPO-Rich and OPO-Rich Lipids

PPP-rich lipid (213.18 g) and oleic acid (219.9 g) (1:3 molar ratio) were completely melted in an Erlenmeyer flask with a lid and placed in a water bath (>80 °C). Lipozyme RMIM (10% of substrate, *w*/*w*) and *n*-hexane (50 mL) were added, and the lipase-catalyzed acidolysis reaction was performed in a shaking water bath (Daihan Labtech Co., Namyangju, Korea) at 200 rpm and 55 °C for 6 h. After removing the enzyme by filtration, the reaction mixture was deacidified with 2 N KOH and 0.1% phenolphthalein, concentrated using a vacuum evaporator, and dried with N_2_. The deacidified lipid was fractionated with acetone (1:5, *w*/*v*) at 9 °C for 24 h and separated into solid and lipid phases. The liquid phase was mixed again with acetone (1:5, *w*/*v*) and fractionated at 5 °C for 24 h. After centrifugation (3134× *g*, 5 min, and 5 °C), the solid phase (PPO-rich lipid) and liquid phase (OPO-rich lipid) were obtained, concentrated using a vacuum evaporator, and dried with N_2._ MAG and DAG were removed from the lipids by washing with methanol (30 min, repeated six times), and TAG was obtained. The purity of the TAG was confirmed by thin-layer chromatography (TLC) using *n*-hexane: diethyl ether: acetic acid (90:10:1, *v*/*v*/*v*).

### 3.4. Analysis of Acylglycerol Composition with ^1^H-Nuclear Magnetic Resonance (NMR)

Lipids (10 mg) were dissolved in 0.1% TMS in CDCl_3_ (100 μL) and placed in a 5 mm NMR tube (Norell, Landisville, NJ, USA). The acylglycerol composition was analyzed using a Bruker Advance III 600 MHz NMR spectrometer (Bruker Corporation, Billerica, MA, USA), and the signals of TAG, DAG, MAG, and TMS from ^1^H-NMR spectra were identified [31,32,33,34]. TMS was used as a reference to calculate the TAG, DAG, and MAG of each lipid in μmol or mmol% units.

### 3.5. Analysis of TAG Composition with Silver Ion-High Performance Liquid Chromatography (Ag-HPLC)

TAGs of the lipids were separated using an Ag-HPLC (Younglin, Anyang, Korea) equipped with a silver ion column (Chromspher 5 lipids, 250 × 4.6 mm i.d., 5.0 μm, Varian, Middleburg, Netherlands) and an evaporative light scattering detector (ELSD, Sedex 75, Sedere, Alfortville, France). The temperature of the ELSD was set at 40 °C, and N_2_ was used as a nebulizing gas at a pressure of 2.2 bar. Lipids were dissolved in chloroform: *n*-hexane (1:1, *v*/*v*) and 20 μL were injected into the HPLC system. The mobile phases were solvent A (*n*-hexane: isopropanol: acetonitrile = 100:0.1:0.1, *v*/*v*/*v*) and solvent B (*n*-hexane: iso-propanol: acetonitrile = 100:1:1, *v*/*v*/*v*) at a flow rate of 1.5 mL/min. TAG separation was started with solvent A for 5 min; the solvent ratio was increased to 80:20 (*v*/*v*) for 10 min, to 50:50 (*v*/*v*) for 10 min, held for 1 min, then returned to the initial condition of solvent A (100%), and finally held for 8 min. The TAGs were separated by the number of double bonds and the position of double bonds in the acyl chains, and the regioisomeric and enantiomeric isomers [37]. The individual TAG peaks in the HPLC chromatogram were identified by standard PPP, and designated by comparing the Ag-HPLC peaks of the OPO human milk fat substitute reported in Lee et al. [13], and TAG species reported in Hong et al. [38], which presented the Ag-HPLC chromatographs with the same methodology as the present study. Each TAG was presented with HPLC peak area or percentage (%) of total TAGs area.

### 3.6. Analysis of Fatty Acid Composition

The lipid (25 mg) was mixed with 0.5 N methanolic NaOH (1.5 mL) and saponified at 85 °C for 10 min. After cooling, BF_3_-methanol (2 mL) was added, and methylated at 85 °C for 10 min. The FA composition was analyzed using a gas chromatography (GC-2010 Plus, Shimadzu Corp., Kyoto, Japan) equipped with a flame ionization detector, and an SP^TM^-2560 capillary column (100 m × 0.25 mm × 0.2 μm film thickness, Supelco Inc., Bellefonte, PA, USA) [39]. The oven temperature was held at 100 °C for 5 min, increased to 240 °C at 4 °C/min, and maintained for 40 min. The temperatures of the injector and detector were 250 and 260 °C, respectively, and the carrier gas and column flow were N_2_ and 1 mL/min, respectively. Triundecanoin was used as an internal standard, and Supelco 37 component FAME mix was used as an external standard. After obtaining the GC chromatogram, each FA was identified by comparison with the peaks of the Supelco 37 component FAME mix and the area of each peak was expressed as a percentage of the total FAs.

For analysis of the positional FA composition, lipid (25 mg), pancreatic lipase (25 mg), 0.05% bile salt (6.25 mL), 2.2% CaCl_2_ (2.5 mL), and Tris-HCl buffer (pH 7.6, 25 mL) were mixed in a test tube and reacted for 3 min at 37 °C, with 30-s vortexing, and repeated three times. Then, 6 mL of diethyl ether were mixed for 1 min and centrifuged (1224× *g*, 5 min). The supernatant was concentrated with N_2_ and the hydrolyzed lipid was separated into TAG, 1,3-DAG, 1,2-DAG, 1-MAG, and 2-MAG on a TLC F_254_ silica plate (Merck, Kenilworth, NJ, USA) with a developing solvent (*n*-hexane: diethyl ether: acetic acid = 50:50:1, *v*/*v*/*v*). The band presenting 2-MAG was taken from the TLC plate, and was saponified and methylated. The FA composition of 2-MAG was analyzed with GC, and the FA composition of the *sn*-1,3 position was calculated with Equation (1):

FA composition at *sn*-1,3 position (%) = [3 × total FA composition (%) − FA composition at *sn*-2(%)]/2. (1)

### 3.7. Analysis of Melting and Crystallization Properties with Differential Scanning Calorimetry (DSC)

The SMPs and CMPs of the lipids were measured using the capillary tube method of the AOCS Official Method Cc 1-25 [40]. The melting and crystallization properties were analyzed using DSC (DSC-8000, Perkin Elmer, Waltham, MA, USA). The sample was accurately weighed (7 ± 0.1 mg) on a DSC aluminum pan, and an empty aluminum pan was used as a reference. The sample was heated to 80 °C and held for 10 min. The crystallization thermogram was obtained by cooling to −60 °C at a rate of 10 °C/min, and after holding for 10 min, the melting thermogram was obtained by heating to 80 °C at a rate of 5 °C/min. All samples were analyzed in triplicate.

### 3.8. Crystal Morphology by Polarized Light Microscopy

The completely melted lipid (20 μL) was placed on a preheated microscope glass slide and covered with a preheated cover slip. For crystallization of the lipid, the prepared slides were stored at 4 °C for 16 h. The crystal morphology was observed using polarized light microscopy (Eclipase 50i POL, Nikon, Tokyo, Japan) and a digital camera (ISH 300, Tucsen Photonics Co., Ltd., Fuzhou, China).

### 3.9. In Vitro Multi-Step Digestion Model

The in vitro digestion degree of lipids was assessed using a modified method described by Versantvoort et al. [41] and Chang et al. [18] (Table 7). All simulated digestive juices were prepared and used on the day of the experiment, and their compositions are presented in Table 7. The completely melted lipid (100 mg) and saliva juice (1.2 mL) were mixed in an Erlenmeyer flask in a shaking water bath for 5 min at 37 °C and 80 rpm, and gastric juice (2.4 mL) was added and allowed to react for 2 h. NaHCO_3_ solution (0.4 mL), bile juice (1.2 mL), and duodenal juice (2.4 mL) were added, and the mixture was hydrolyzed for 30, 60, and 120 min. After the reaction, a lipase inhibitor (100 μL, 0.2 g of 4-bromophenylboronic acid/mL in methanol) was added to stop digestion [42]. For the extraction of hydrolyzed lipids, *n*-hexane was added, mixed, and centrifuged at 1763× *g* for 5 min, and the supernatant was collected (repeated three times). Next, 1 N HCl (0.5 mL) was mixed with the remaining lower part for 1 min, then *n*-hexane (10 mL) was mixed and centrifuged, and the supernatant was collected (repeated three times). The all *n*-hexane extraction was combined, and then the solvent was removed by using N_2_. Next, 10 mL of ethanol: *n*-hexane (1:1, *v*/*v*) and 1 mL of 1% phenolphthalein in ethanol were added and titrated with 0.05 N KOH solution. The in vitro digestion degree of each lipid was expressed as the released FFA (%) using Equation (2) [43]:

(2) $$\mathrm{FFAs}\;\mathrm{released}\;\left(\%\right)=\frac{\mathrm{Volume}\;\mathrm{of}\;\mathrm{KOH}\;\left(\mathrm{mL}\right)\;\times \;\mathrm{Normality}\;\mathrm{of}\;\mathrm{KOH}\;\left(N\right)\;\times \mathrm{Molecular}\;\mathrm{weight}\;\mathrm{of}\;\mathrm{lipid}}{\mathrm{Amount}\;\mathrm{of}\;\mathrm{lipid}\;\mathrm{used}\;\mathrm{digestion}\;\left(\mathrm{mg}\right)\;\times 2}\times 100$$

### 3.10. Statistical Analysis

Analysis of variance was performed using the Statistical Analysis System 9.4 (SAS Institute, Cary, NC, USA). The statistical significance of the experimental means was analyzed by Duncan’s multiple range test and Student’s *t*-test. A *p* value < 0.05 was considered significant.

## 4. Conclusions

PPP-rich, POP-rich, PPO-rich, and OPO-rich lipids with different regiospecific positions of palmitic acid were synthesized, and their physicochemical and digestion characteristics were compared. The PPP-rich, POP-rich, PPO-rich, and OPO-rich lipids contained PPP (81.2%), POP (64.4%), PPO (86.5%), and OPO (50.2%), respectively, as the major TAG. POP-rich and PPO-rich lipids have similar total fatty acid composition; however, the POP-rich lipid had a higher palmitic acid content at the *sn*-1,3 position, and higher melting points, crystallization temperatures, and packing densities of fat crystals than PPO-rich lipid. The in vitro digestion degree analyzed by the released FA(%) at 30, 60, and 120 min was followed by OPO-rich > PPO-rich > POP-rich lipids. As an individual TAG, POP (in POP-rich lipid) was hydrolyzed faster than PPO (in PPO-rich lipid) and OPO (in OPO-rich lipid), showing the digestion extent by 92.3%, 89.7%, and 87.2%, respectively. This study confirmed that the content and incorporated position of palmitic acid in the TAG backbone affect the physicochemical and digestion properties. In addition, based on the established in vitro multi-step model for assessment of the digestion properties of specific TAG-enriched lipids in this study, an in vitro absorption study that predicts the absorption characteristics of lipids using Caco-2 cells will be required in the future.

## Figures and Tables

**Table 1:** Acylglyerol composition of the lipids.

Acylglycerol (mmol%)	Palm Stearin	PPP-Rich Lipid	POP-Rich Lipid	PPO-Rich Lipid	OPO-Rich Lipid
Triacylglycerol (TAG)	96.9 ± 0.0 ^d(1)^	100.0 ± 0.0 ^a^	93.4 ± 0.0 ^e^	99.1 ± 0.0 ^c^	99.3 ± 0.0 ^b^
Diacylglycerol (DAG)	3.1 ± 0.0 ^b^	0.0 ± 0.0 ^d^	6.3 ± 0.1 ^a^	0.7 ± 0.0 ^c^	0.7 ± 0.0 ^c^
1,3-DAG	1.3 ± 0.0 ^b^	0.0 ± 0.0 ^d^	3.1 ± 0.1 ^a^	0.4 ± 0.0 ^c^	0.3 ± 0.0 ^c^
1,2-DAG	1.9 ± 0.0 ^b^	0.0 ± 0.0 ^d^	3.3 ± 0.1 ^a^	0.3 ± 0.0 ^c^	0.3 ± 0.0 ^c^
Monoacylglycerol (MAG)	- ^(2)^	-	0.3 ± 0.0 ^a^	0.2 ± 0.0 ^b^	0.1 ± 0.0 ^c^
1-MAG	-	-	0.1 ± 0.0 ^b^	0.2 ± 0.0 ^a^	0.0 ± 0.0 ^c^
2-MAG	-	-	0.1 ± 0.0 ^a^	0.0 ± 0.0 ^b^	0.1 ± 0.0 ^b^

^(1) a–e^ Means in the same row with different letters are significantly different according to Duncan’s multiple range test at *p* < 0.05. Mean ± SD (*n* = 2). ^(2)^: Not detected.

**Table 2:** Total fatty acids composition and melting points of the lipids.

Fatty Acid (% of Total Fatty Acids)	Palm Stearin	PPP-Rich Lipid	POP-Rich Lipid	PPO-Rich Lipid	OPO-Rich Lipid
C14:0	1.1 ± 0.0	1.3 ± 0.0	0.9 ± 0.0	0.5 ± 0.0	0.7 ± 0.0
C16:0	63.4 ± 0.2 ^b(1)^	79.4 ± 0.0 ^a^	46.5 ± 0.0 ^d^	46.9 ± 0.0 ^c^	30.2 ± 0.1 ^e^
C18:0	4.8 ± 0.0 ^b^	5.0 ± 0.0 ^a^	4.5 ± 0.0 ^c^	3.7 ± 0.0 ^d^	2.5 ± 0.1 ^e^
C18:1t	0.1 ± 0.0	- ^(2)^	0.1 ± 0.0	0.7 ± 0.0	1.0 ± 0.0
C18:1n-9c	24.9 ± 0.2 ^d^	11.6 ± 0.0 ^e^	38.9 ± 0.0 ^c^	45.3 ± 0.0 ^b^	60.9 ± 0.2 ^a^
C18:1n-7c	0.4 ± 0.0	0.2 ± 0.0	0.6 ± 0.0	-	-
C18:2n-6c	5.1 ± 0.0 ^b^	2.1 ± 0.0 ^e^	8.2 ± 0.0 ^a^	2.8 ± 0.0 ^d^	4.6 ± 0.1 ^c^
C20:0	0.3 ± 0.0	0.3 ± 0.0	0.3 ± 0.0	0.2 ± 0.0	0.2 ± 0.0
SFA ^(3)^	69.6 ± 0.2 ^b^	86.0 ± 0.0 ^a^	52.2 ± 0.0 ^c^	51.2 ± 0.0 ^d^	33.5 ± 0.2 ^e^
USFA ^(3)^	30.4 ± 0.2 ^d^	14.0 ± 0.0 ^e^	47.8 ± 0.0 ^c^	48.8 ± 0.0 ^b^	66.5 ± 0.2 ^a^
MUFA ^(3)^	25.3 ± 0.2 ^d^	11.9 ± 0.0 ^e^	39.6 ± 0.0 ^c^	46.0 ± 0.0 ^b^	61.9 ± 0.3 ^a^
Slip melting point (°C)	55.6 ± 0.4 ^b^	59.5 ± 0.7 ^a^	27.3 ± 0.4 ^c^	26.5 ± 0.0 ^c^	15.0 ± 0.0 ^d^
Complete melting point (°C)	57.0 ± 0.0 ^b^	61.5 ± 0.0 ^a^	29.5 ± 0.7 ^c^	27.5 ± 0.0 ^d^	18.5 ± 0.7 ^e^

^(1) a–e^ Means in the same row with different letters are significantly different at *p* < 0.05, as determined by Duncan’s multiple range test. Mean ± SD (*n* = 2). ^(2)^: Not detected. ^(3)^ SFA, saturated fatty acid; USFA, unsaturated fatty acid; MUFA, monounsaturated fatty acid.

**Table 3:** The *sn*-2 and *sn*-1, 3 positional fatty acid compositions of the lipids.

Fatty Acid (% of Total Fatty Acids)	Palm Stearin	PPP-Rich Lipid	POP-Rich Lipid	PPO-Rich Lipid	OPO-Rich Lipid
*sn*-2 position					
C16:0	45.6 ± 1.6 ^d(1)^	73.4 ± 0.3 ^b^	14.3 ± 0.1 ^e^	81.2 ± 2.1 ^a^	60.3 ± 0.7 ^c^
C18:0	2.8 ± 0.8 ^ab^	4.3 ± 1.5 ^a^	1.3 ± 0.0 ^b^	2.2 ± 0.3 ^b^	3.1 ± 0.0 ^ab^
C18:1n-9c	42.5 ± 1.8 ^b^	19.9 ± 0.2 ^d^	67.9 ± 0.1 ^a^	13.0 ± 1.0 ^e^	31.8 ± 0.1 ^c^
C18:2n-6c	9.1 ± 0.6 ^b^	2.3 ± 0.9 ^d^	16.5 ± 0.0 ^a^	3.6 ± 0.8 ^cd^	4.8 ± 0.8 ^c^
SFA ^(2)^	48.4 ± 2.4 ^d^	77.8 ± 1.2 ^b^	15.7 ± 0.1 ^e^	83.4 ± 1.8 ^a^	63.4 ± 0.7 ^c^
USFA ^(2)^	51.6 ± 2.4 ^b^	22.2 ± 1.2 ^d^	84.3 ± 0.1 ^a^	16.6 ± 1.8 ^e^	36.6 ± 0.7 ^c^
MUFA ^(2)^	42.5 ± 1.8 ^b^	19.9 ± 0.2 ^d^	67.9 ± 0.1 ^a^	13.0 ± 1.0 ^e^	31.8 ± 0.1 ^c^
*sn*-1,3 position					
C14:0	1.6 ± 0.0	1.9 ± 0.0	1.3 ± 0.0	0.8 ± 0.0	1.0 ± 0.0
C16:0	72.3 ± 0.4 ^b^	82.3 ± 0.2 ^a^	62.5 ± 0.0 ^c^	29.7 ± 1.1 ^d^	15.2 ± 0.6 ^e^
C18:0	5.8 ± 0.5 ^a^	5.4 ± 0.7 ^ab^	6.1 ± 0.0 ^a^	4.5 ± 0.2 ^b^	2.2 ± 0.1 ^c^
C18:1t	0.1 ± 0.0	- ^(3)^	0.1 ± 0.0	1.1 ± 0.0	1.5 ± 0.1
C18:1n-9c	16.0 ± 0.6 ^d^	7.5 ± 0.1 ^e^	24.3 ± 0.0 ^c^	61.4 ± 0.5 ^b^	75.4 ± 0.3 ^a^
C18:1n-7c	0.6 ± 0.0	0.3 ± 0.0	1.0 ± 0.0	-	-
C18:2n-6c	3.0 ± 0.3 ^b^	2.0 ± 0.5 ^c^	4.1 ± 0.0 ^a^	2.3 ± 0.4 ^bc^	4.5 ± 0.3 ^a^
C20:0	0.5 ± 0.0	0.5 ± 0.0	0.5 ± 0.0	0.2 ± 0.0	0.2 ± 0.0
SFA ^(2)^	80.2 ± 0.9 ^b^	90.1 ± 0.6 ^a^	70.4 ± 0.0 ^c^	35.2 ± 0.9 ^d^	18.6 ± 0.7 ^e^
USFA ^(2)^	19.8 ± 0.9 ^d^	9.9 ± 0.6 ^e^	29.6 ± 0.0 ^c^	64.8 ± 0.9 ^b^	81.4 ± 0.7 ^a^
MUFA ^(2)^	16.8 ± 0.6 ^d^	7.8 ± 0.1 ^e^	25.4 ± 0.0 ^c^	62.5 ± 0.5 ^b^	76.9 ± 0.3 ^a^

^(1) a–e^ Means in the same row with different letters are significantly different at *p* < 0.05, as determined by Duncan’s multiple range test. Mean ± SD. (*n* = 2). ^(2)^: SFA, saturated fatty acid; USFA, unsaturated fatty acid; MUFA, monounsaturated fatty acid. ^(3)^: Not detected.

**Table 4:** Triacylglycerol compositions of lipids.

Triacylglycerol ^(1)^ (% of Total Triayclglyerol)	Palm Stearin	PPP-Rich Lipid	POP-Rich Lipid	PPO-Rich Lipid	OPO-Rich Lipid
SaSaSa (PPP)	38.0 ± 0.2 ^b(2)^	81.2 ± 1.1 ^a^	0.6 ± 0.0 ^c^	- ^(3)^	-
SaMoSa (POP)	42.4 ± 0.3 ^b^	15.1 ± 0.8 ^c^	64.4 ± 1.4 ^a^	0.7 ± 0.1 ^d^	1.6 ± 0.1 ^d^
SaSaMo (PPO)	3.8 ± 0.2 ^c^	1.2 ± 0.1 ^d^	5.5 ± 0.0 ^c^	86.5 ± 0.2 ^a^	22.0 ± 2.0 ^b^
SaDSa (PLP)	3.2 ± 0.1^b^	0.8 ± 0.1 ^c^	5.8 ± 0.2 ^a^	0.5 ± 0.0 ^d^	0.2 ± 0.0 ^e^
SaSaD/SaMoMo (PPL/POO)	-	-	-	2.9 ± 0.1	18.6 ± 0.4 *^(4)^
MoSaMo (OPO)	10.8 ± 0.3 ^c^	1.9 ± 0.1 ^d^	20.3 ± 0.9 ^b^	8.7 ± 0.1 ^c^	50.2 ± 2.7 ^a^
SaDMo/SaMoD (PLO/POL)	1.5 ± 0.1 ^b^	-	2.9 ± 0.4 ^a^	-	0.7 ± 0.1 ^c^
MoMoMo (OOO)	0.3 ± 0.0 ^d^	-	0.6 ± 0.0 ^c^	0.8 ± 0.1^b^	6.8 ± 0.1 ^a^

^(1)^ The abbreviations were presented in the results and discussion, and abbreviations in parentheses refer to the major TAGs. ^(2) a–e^: Means in the same row with different letters are significantly different at *p* < 0.05, as determined by Duncan’s multiple range test. Mean ± SD. (*n* = 2). ^(3)^: Not detected. ^(4)^ *: Differences between PPO-rich lipid and OPO-rich lipid are significantly different by Student’s *t*-test (*p* < 0.05).

**Table 5:** Acylglycerol compositions of POP-rich lipid, PPO-rich lipid, and OPO-rich lipids determined by the in vitro multi-step digestion model.

Acylglycerol (μmol)	POP-Rich Lipid			PPO-Rich Lipid			OPO-Rich Lipid		
	Before Digestion	In Vitro Digestion		Before Digestion	In Vitro Digestion		Before Digestion	In Vitro Digestion	
		60 min	120 min		60 min	120 min		60 min	120 min
Triacylglycerol (TAG)	810.76 ± 12.27 ^a(1)^ (100%) ^(2)^	158.33 ± 6.17 ^b^ (19.53%)	97.89 ± 1.80 ^c^ (12.10%)	848.12 ± 58.08 ^a^ (100%)	130.04 ± 1.23 ^b^ (15.33%)	101.04 ± 1.80 ^b^ (11.91%)	727.01 ± 31.00 ^a^ (100%)	100.73 ± 0.38 ^b^ (13.86%)	74.20 ± 3.38 ^b^ (10.21%)
Diacylglycerol (DAG)	54.96 ± 1.52 ^b^ (100%)	68.97 ± 3.69 ^a^ (125.49%)	32.51 ± 4.06 ^c^ (59.15%)	6.12 ± 1.72 ^b^ (100%)	54.49 ± 5.80 ^a^ (890.36%)	45.07 ± 2.79 ^a^ (736.44%)	4.79 ± 0.14 ^c^ (100%)	40.62 ± 0.93 ^a^ (848.02%)	15.24 ± 2.65 ^b^ (318.16%)
1,3-DAG	26.55 ± 1.66 ^a^	15.67 ± 1.86 ^b^	8.52 ± 0.02 ^c^	3.57 ± 0.46 ^b^	12.87 ± 1.68 ^a^	12.22 ± 0.26 ^a^	2.48 ± 0.05 ^b^	12.21 ± 0.32 ^a^	3.25 ± 0.42 ^b^
1,2-DAG	28.41 ± 0.14 ^b^	53.30 ± 1.83 ^a^	22.99 ± 4.03 ^b^	2.56 ± 0.23 ^b^	41.63 ± 4.12 ^a^	32.85 ± 3.06 ^a^	2.31 ± 0.18 ^c^	28.41 ± 0.61 ^a^	11.99 ± 2.22 ^b^
Monoacylglycerol (MAG)	2.30 ± 0.26 ^a^ (100%)	2.43 ± 0.22 ^a^ (105.65%)	3.21 ± 0.86 ^a^ (139.57%)	1.58 ± 0.01 ^b^ (100%)	4.20 ± 0.81 ^a^ (265.82%)	2.86 ± 0.34 ^ab^ (181.01%)	0.58 ± 0.08 ^b^ (100%)	5.82 ± 1.79 ^a^ (1003.45%)	3.84 ± 0.11 ^ab^ (662.07%)
1-MAG	1.08 ± 0.03 ^a^	1.36 ± 0.18 ^a^	1.85 ± 0.50 ^a^	1.25 ± 0.06 ^b^	2.65 ± 0.67 ^a^	1.68 ± 0.05 ^ab^	0.15 ± 0.01 ^b^	3.64 ± 1.27 ^a^	1.87 ± 0.29 ^ab^
2-MAG	1.22 ± 0.22 ^a^	1.07 ± 0.05 ^a^	1.37 ± 0.36 ^a^	0.33 ± 0.05 ^b^	1.55 ± 0.14 ^a^	1.18 ± 0.29 ^a^	0.43 ± 0.07 ^b^	2.18 ± 0.52 ^a^	1.97 ± 0.18 ^a^

^(1) a–c^ Mean in the same row with different letters are significantly different in each lipid at *p* < 0.05 according to Duncan’s multiple range test. ^(2)^ The value (%) in parentheses is the relative ratio based on before digestion.

**Table 6:** Triacylglycerol compositions of POP-rich, PPO-rich, and OPO-rich lipids during in vitro multi-step digestion analyzed with Ag-HPLC.

Triacylglycerol ^(1)^ (Area of Total Triacylglycerol)	POP-Rich Lipid				PPO-Rich Lipid				OPO-Rich Lipid			
	Before Digestion	In Vitro Digestion			Before Digestion	In Vitro Digestion			Before Digestion	In vitro Digestion		
		30 min	60 min	120 min		30 min	60 min	120 min		30 min	60 min	120 min
SaSaSa (PPP)	140 ± 18 ^(2)^	- ^(3)^	-	-	-	-	-	-	-	-	-	-
SaMoSa (POP)	16493 ± 3410 ^a(4)^ (100%) ^(5)^	7180 ± 2538 ^b^ (43.53%)	3458 ± 1772 ^b^ (20.97%)	1705 ± 278 ^b^ (10.34%)	284 ± 4 ^a^ (100%)	43 ± 16 ^b^ (15.14%)	40 ± 9 ^b^ (14.08%)	54 ± 9 ^b^ (19.01%)	339 ± 55 *^(6)^ (100%)	28 ± 1 (8.26%)	-	-
SaSaMo (PPO)	1403 ± 261 ^a^ (100%)	457 ± 196 ^b^ (32.57%)	132 ± 97 ^b^ (9.41%)	69 ± 11 ^b^ (4.92%)	37079 ± 3403 ^a^ (100%)	5855 ± 2899 ^b^ (15.79%)	5977 ± 188 ^b^ (16.12%)	4730 ± 3071 ^b^ (12.76%)	4756 ± 594 ^a^ (100%)	416 ± 70 ^b^ (8.75%)	289 ± 23 ^b^ (6.08%)	187 ± 28 ^b^ (3.93%)
SaDSa (PLP)	1468 ± 222 ^a^ (100%)	610 ± 202 ^b^ (41.55%)	258 ± 141 ^b^ (17.57%)	142 ± 59 ^b^ (9.67%)	211 ± 18 ^a^ (100%)	29 ± 3 ^b^ (13.74%)	36 ± 9 ^b^ (17.06%)	27 ± 3 ^b^ (12.80%)	37 ± 7	-	-	-
SaSaD/SaMoMo (PPL/POO)	-	-	-	-	1226 ± 145 ^a^ (100%)	195 ± 17 ^b^ (15.91%)	169 ± 15 ^b^ (13.78%)	160 ± 37 ^b^ (11.70%)	4047 ± 800 ^a^ (100%)	432 ± 263 ^b^ (10.67%)	332 ± 48 ^b^ (8.20%)	178 ± 43 ^b^ (4.40%)
MoSaMo (OPO)	5172 ± 741 ^a^ (100%)	2077 ± 763 ^b^ (40.16%)	830 ± 610 ^b^ (16.05%)	523 ± 155 ^b^ (10.11%)	3728 ± 278 ^a^ (100%)	550 ± 136 ^b^ (14.75%)	548 ± 68 ^b^ (14.70%)	436 ± 262 ^b^ (11.70%)	11,019 ± 2963 ^a^ (100%)	1088 ± 124 ^b^ (9.87%)	803 ± 222 ^b^ (7.29%)	411 ± 124 ^b^ (3.73%)
SaDMo/SaMoD (PLO/POL)	720 ± 42	-	-	-	-	-	-	-	155 ± 6	-	-	-
MoMoMo (OOO)	149 ± 37	-	-	-	347 ± 4	-	-	-	1482 ± 291	-	-	-
Unknown	-	3457 ± 1027 ^a^	5244 ± 578 ^a^	6898 ± 1633 ^a^	-	1004 ± 429 ^b^	2606 ± 216 ^ab^	4444 ± 1215 ^a^	-	1148 ± 86 ^b^	1955 ± 287 ^ab^	4564 ± 1601 ^a^
FFA ^(7)^	-	4678 ± 368 ^b^	5515 ± 1139 ^b^	8486 ± 255 ^a^	-	11,154 ± 3465 ^a^	12,298 ± 714 ^a^	15,923 ± 688 ^a^	-	18,293 ± 2863 ^b^	26,264 ± 4074 ^ab^	30,650 ± 4044 ^a^

^(1)^ The abbreviations were presented in the results and discussion, and abbreviations in parentheses refer to the major TAG. ^(2)^ Mean ± SD. (*n* = 2). ^(3)^: Not detected. ^(4) a–c^ Mean in the same row with different letters are significantly different in each lipid by Duncan’s multiple range test (*p* < 0.05). ^(5)^ The values (%) in parentheses are relative area ratio based on before digestion. ^(6)^ *: Differences between before digestion and 30 min-digested lipid are significantly different by Student’s *t*-test (*p* < 0.05). ^(7)^ FFA: Free fatty acids.

**Table 7:** The composition of synthetic juices for the in vitro multi-step digestion model ^(1)^.

	Saliva Juice	Gastric Juice	Duodenal Juice	Bile Juice
Inorganic solution	1 mL KCl (89.6 g/L)	1.57 mL NaCl (175.3 g/L)	4 mL NaCl (175.3 g/L)	3 mL NaCl (175.3 g/L)
	1 mL KSCN (20 g/L)	0.3 mL NaH_2_PO_4_ (88.8 g/L)	4 mL NaHCO_3_ (84.7 g/L)	6.83 mL NaHCO_3_ (84.7 g/L)
	1 mL NaH_2_PO_4_ (88.8 g/L)	0.92 mL KCl (89.6 g/L)	1 mL KH_2_PO_4_ (8 g/L)	0.42 mL KCl (89.6 g/L)
	0.17 mL NaCl (175.3 g/L)	1.8 mL CaCl_2_·2H_2_O (22.2 g/L)	0.63 mL KCl (89.6 g/L)	1 mL CaCl_2_·2H_2_O (22.2 g/L)
	2 mL NaHCO_3_ (84.7 g/L)	1 mL NH_4_Cl (30.6 g/L)	1 mL MgCl_2_ (5 g/L)	15 μL HCl (440.3 g/L)
		0.65 mL HCl (440.3 g/L)	0.9 mL CaCl_2_·2H_2_O (22.2 g/L)	
			18 μL HCl (440.3 g/L)	
Organic solution		1 mL Glucose (65 g/L)		
	0.8 mL Urea (25 g/L)	1 mL Glucuronic acid (2 g/L)	0.4 mL Urea (25 g/L)	1 mL Urea (25 g/L)
		0.34 mL Urea (25 g/L)		
		1 mL Glucosamine (33 g/L)		
Supplementation to the solution	α-amylase 29 mg	BSA 0.1 g	BSA 0.1 g	BSA 0.18 g
	Uric acid 1.5 mg	Pepsin 0.25 g	Pancreatin 0.9 g	Bile salt 3 g
	Mucin 2.5 mg	Mucin 0.3 g	Lipase 0.15 g	
pH	6.8 ± 0.2 ^(2)^	1.3 ± 0.02	8.1 ± 0.2	8.2 ± 0.2

^(1)^ Versantvoort et al. [41]; Chang et al. [18]. ^(2)^ Mean ± SD.

## Data Availability

Not applicable.

## Sample Availability

Samples of the compounds are not available from the authors.
